# Supplementary material for: MetaRibo-Seq measures translation in microbiomes
Source: Nat Commun. 2020 Jun 29;11:3268. doi: 10.1038/s41467-020-17081-z (PMC7324362; doi:10.1038/s41467-020-17081-z)
Supplement: Supplementary file 10 — Supplementary Data 7 [file 41467_2020_17081_MOESM10_ESM.zip › File2/Confidence_High_Taxonomy/351938_out.krona.html]

Javascript must be enabled to view this page.

members
magnitude
magnitudeUnassigned
count
unassigned
taxon
rank

351938\_out

3

2
3
superkingdom

3
1239
phylum

186801
3
class

3
186802
order

541000
3
family

genus
1
216851

species

SRS015890\_contig\_number\_10560
1
853


SRS054059\_contig\_number\_88SRS147271\_contig\_number\_38875
species
1898205
2
